# Neuroplasticity and the microbiome: how microorganisms influence brain change

**DOI:** 10.3389/fmicb.2025.1629349

**Published:** 2025-08-20

**Authors:** Abdullah Al Noman, Abdulrahman Mohammed Alhudhaibi, Moushumi Afroza, Susmita Deb Tonni, Habibul Mohsin Shehab, Nusrat Jahan Iba, Tarek H. Taha, Emad M. Abdallah

**Affiliations:** ^1^School of Pharmacy, BRAC University, Dhaka, Bangladesh; ^2^Department of Biology, College of Science, Imam Mohammad Ibn Saud Islamic University (IMSIU) Riyadh, Saudi Arabia; ^3^Department of Biological Science, St. John's University, Queens, NY, United States; ^4^Department of Pharmacy, State University of Bangladesh, Dhaka, Bangladesh; ^5^Department of Biology, College of Science, Qassim University, Buraydah, Saudi Arabia

**Keywords:** neuroplasticity, gut-brain axis, microbiota, cognitive function, health

## Abstract

Neuroplasticity, the brain's ability to reorganize and adapt, has traditionally been attributed to external stimuli, learning, and experience. However, emerging research highlights the gut microbiota as a key modulator of neuroplasticity through the gut-brain axis. This review examines the mechanisms by which intestinal microorganisms influence brain function, including microbial metabolite production, immune system modulation, neurotransmitter synthesis, and hormonal regulation. Dysbiosis, or microbial imbalance, has been linked to neurodevelopmental disorders, major depressive disorder (MDD), and cognitive impairments, emphasizing the microbiome's role in psychiatric and neurological health. Animal and human studies suggest that microbiota-targeted interventions, such as probiotics, prebiotics, and dietary modifications, may enhance neuroplasticity and mitigate mental health disorders. Future research should focus on understanding direct microbial-neuronal interactions and developing personalized microbiome-based therapies. This study underscores the gut microbiota's potential as a novel target for improving brain plasticity and mental health outcomes.

## 1 Introduction

Neuroplasticity alludes to the capacity of brain circuits to reorganize and alter the properties of the arrangement coming about in modifications in brain work and behavior (Zilles, 1992). It is customarily accepted that neuroplasticity is impacted by outside jolts learning and encounters (Damiani et al., 2023). Intriguingly there's modern proof proposing that endogenous signals from the body's outskirts may play a part. The intestine microbiota a different community of microorganisms living in agreement with their have may be able to impact plasticity through its balance of the gut-brain pivot (Bercik et al., 2011). Interest in the development of the intestine microbiota coincides with basic periods of neurodevelopment amid which neural circuits are exceedingly plastic and possibly defenseless. As such dysbiosis, and an awkwardness within the intestine microbiota composition amid early life may contribute to the disturbance of ordinary formative directions driving to neurodevelopmental clutters. This survey points to look at how the intestine microbiota can influence neuroplasticity (Suda and Matsuda, 2022). The intestinal microbiota influences brain chemistry and behavior independently of the autonomic nervous system, gastrointestinal-specific neurotransmitters, or inflammation. Intestinal dysbiosis might contribute to psychiatric disorders in patients with bowel disorders (Bercik et al., 2011). Neuroplasticity alludes to the capacity of brain circuits to reorganize and alter the properties of the arrange coming about in modifications in brain work and behavior (Zilles, 1992). It is customarily accepted that neuroplasticity is impacted by outside stimuli learning and encounters (Zilles, 1992). Intriguingly there's unused prove recommending that endogenous signals from the body outskirts may play a part. The intestine microbiota a different community of microorganisms living in concordance with their have may be able to impact versatility through its tweak of the gut-brain pivot (Ganguly and Poo, 2013). Interests in the development of the intestine microbiota coincide with basic periods of neurodevelopment amid which neural circuits are profoundly plastic and possibly powerless (Fuchs and Flügge, 2014). As such dysbiosis an imbalance within the intestine microbiota composition amid early life may contribute to the disturbance of ordinary formative directions driving neurodevelopmental disarranges (Kelly et al., 2017).

Major depressive clutter MDD features a tall predominance and may be a major supporter of the worldwide burden of infection (Kelly et al., 2017; Miguel et al., 2019). Emerging research underscores the pivotal role of the gut microbiome in regulating brain function and psychological wellbeing via the gut–brain axis, with disruptions in microbial composition, commonly referred to as gut dysbiosis, being increasingly implicated in neuropsychiatric disorders, including major depressive disorder. Concurrently, an expanding body of evidence highlights the intricate interaction between genetic predispositions and environmental exposures, wherein molecular mechanisms, particularly epigenetic modifications, mediate gene–environment interplay that ultimately shapes individual variability in behavior and health outcome (Boyce et al., 2020; Suda and Matsuda, 2022). A few ponders have detailed that intestine dysbiosis induced aggravation may cause and or contribute to the advancement of discouragement through dysregulation of the gut-brain axis In fact as a result of intestine dysbiosis neuroinflammatory modifications caused by microglial enactment beside disabilities in neuroplasticity may contribute to the improvement of depressive side effects (de Souza et al., 2023). The balance of the intestine microbiota has been recognized as a potentially helpful technique for the administration of MMD In this respect physical workout has appeared to emphatically alter microbiota composition and differing qualities and this will underlie at slightest in portion its upper impacts (Halperin and Healey, 2011; de Souza et al., 2023). The composition of the gut microbiota, the vast community of microorganisms residing in our intestines, plays a crucial role in the management of MDD. Emerging evidence indicates that regular physical exercise can beneficially alter both the diversity and relative abundance of these bacterial populations, potentially accounting for many of the mood-enhancing effects of activity (de Souza et al., 2023; Desbonnet et al., 2015).

This review aims to critically evaluate emerging evidence on the gut microbiome's influence on neuroplasticity through the gut-brain axis, emphasizing mechanisms such as microbial metabolites, immune modulation, neurotransmitter regulation, and hormonal signaling. Drawing from animal and human studies, it explores the impact of gut dysbiosis on neurodevelopmental and psychiatric disorders, including major depressive disorder, and evaluates the therapeutic potential of interventions like probiotics, prebiotics, dietary strategies, and physical activity. The review also highlights key research gaps and proposes future directions for advancing microbiome-based approaches to enhance brain plasticity and mental health.

## 2 Relevant sections

### 2.1 Overview of the gut-brain axis (GBA)

The brain and gastrointestinal tract are interconnected by the convoluted, two-way gut-brain axis (GBA). This eventually impacts negatively on mental and physical health. It is constructed through various parts, which include the vagus nerve, the enteric nervous system (ENS), and microbial metabolites. This system works cooperatively for controlling mood, behavior, digestion, and metabolism. Beyond digestion, the GBA affects immunologic response and, at the same time, response to stress, and neurotransmitters. Comprehending these elements is essential to generate interventions that focus on modifications to lifestyles and mental health (Kodidala et al., 2024).

#### 2.1.1 Key components of the gut-brain axis

(i) Enteric nervous system (ENS): ENS known as the “second brain,” is a massive structure of neurons located in the gut that communicates with the central nervous system (CNS) to control several procedures of the GI tract. This system is responsible for the maintenance of digestive processes and disorders related to neurons also in the gut-brain axis (Nakhal et al., 2024).

(ii) The vagus nerve: The vagus is a cranial nerve that serves as the primary carrier for signaling between the gut and the brain. It regulates mood and stress reactions by transferring signals in the GBA (Kodidala et al., 2024).

(iii) Microbial metabolites: The critical role of the microbiome in the GBA is seen by its generation of metabolites by the gut microbiota, which may involve short-chain fatty acids (SCFAs), that currently have a direct effect on the maintenance of behavior and proper brain function (Bakshi et al., 2024).

In a sense, GBA is often estimated as a pathway for enhancing health, because if disruptions occur in this axis can lead to various disorders- anxiety and depression. This emphasizes several further research studies incorporating therapeutic interventions targeting the GBA to promote mental and physical health.

#### 2.1.2 Communication pathways in the GBA

The ENS and vagus nerve enable the gut and brain to exchange information directly the pathway is known as the neural pathway and this works by influencing the emotional and cognitive areas of the brain (Nakhal et al., 2024).

Another communication route is available, the endocrine pathway, which is closely connected to gut health. This pathway helps to regulate gut hormones and neurotransmitters like serotonin that control mood and appetite. The gut microbiota controls the expression of inflammatory molecules that might affect immunological barrier responses, as well as the GBA directly by the immune-mediated pathway.

The study of neuroplasticity and the intestinal microbiome demonstrates significant challenges and research directions. There are major gaps in understanding the complex interactions between gut microbiota and brain function. There are numerous enhanced approaches and mutually beneficial efforts are required for advancement in the field.

### 2.2 Gut microbiota and neuroplasticity: current understanding

The gut microbiome contains thousands of microbes that are necessary for a number of physiological processes including digestion, metabolism, and immune-mediated control. Recent studies have highlighted the effects it has on the central nervous system (CNS), leading to the concept of the microbiota-gut-brain axis. This interconnection is a bidirectional network that explains that gut health is capable of having an enormous effect on brain health, though the exact mechanisms are still being studied. Some points need to be incorporated in the research part of this concept.

### 2.3 Mechanisms of microbial influence on neuroplasticity

**Figure 1:**
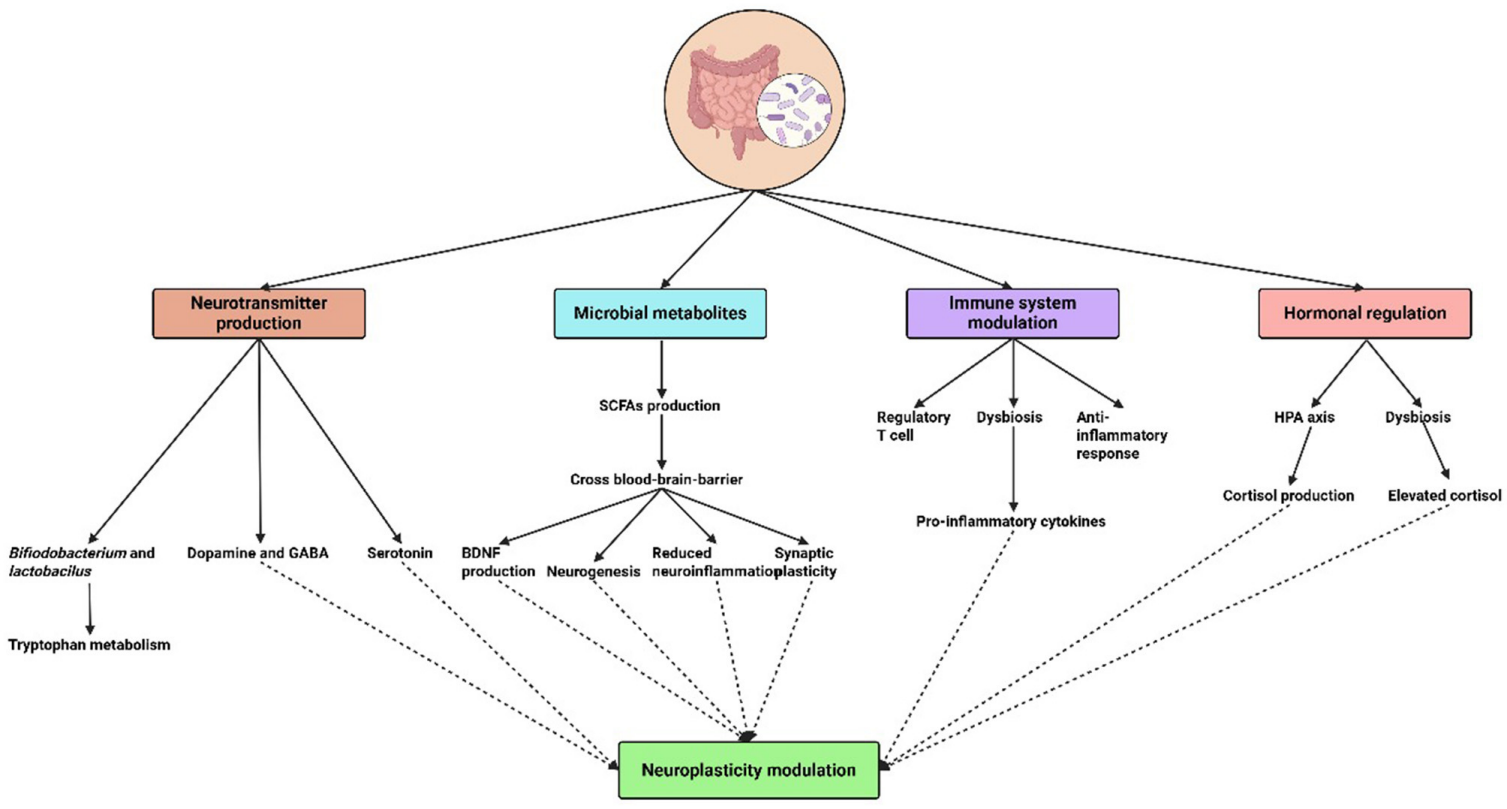
Mechanisms of microbial influence on neuroplasticity.

Neuroplasticity, or the brain's capacity to change and reorganize in response to experience, is increasingly understood to be influenced by the gut microbiota (Damiani et al., 2023). The effects of microbial metabolites, immune system modulation, neurotransmitter synthesis, and hormone regulation are just a few of the domains that are covered in understanding the mechanisms via which the microbiome regulates neuroplasticity (Table 1 and Figure 1).

**Table 1:** Mechanisms of microbial influence on neuroplasticity.

**Mechanism**	**Microbial component**	**Effects on neuroplasticity**	**Examples**	**Reference**
Microbial metabolites	Short-chain fatty acids (SCFAs)	Enhance synaptic plasticity, modulate neurotransmitter release	*Prevotella, Bacteroides, Ruminococcaceae, Lachnospiraceae*	Ju et al., 2023
Immune system modulation	Bacterial cell wall components (e.g., lipopolysaccharide)	Regulate neuroinflammation, promote neurogenesis	*Bacteroides, Firmicutes*	Shi et al., 2020
Neurotransmitter production	Probiotic strains affecting mood and cognition	Alter neurotransmitters like GABA and serotonin, encouraging synaptic plasticity	*Lactobacillus* and *Bifidobacterium*	Huang and Wu, 2021
Hormonal regulation	Stress-regulating bacteria	Balance stress hormones like cortisol	*Lactobacillus reuteri, Bifidobacterium* spp.	Zhang et al., 2023
Neurotrophic factors	Gut bacteria promoting BDNF production	Enhance neurogenesis, synaptic growth	*Akkermansia muciniphila*	Molska et al., 2024

#### 2.3.1 Microbial metabolites and their neuroplastic effects

Gut bacteria degrade food fibers to produce metabolites, including short-chain fatty acids (SCFAs). The blood-brain barrier can be crossed by SCFAs, such as butyrate, propionate, and acetate, which are taken into the bloodstream (Silva et al., 2020). SCFAs have been demonstrated to affect synaptic plasticity, neurogenesis, and brain metabolism once they are inside the brain. In addition to supporting neuronal growth and maturation, SCFAs also regulate neuroinflammatory responses and improve the blood-brain barrier's integrity. According to research, SCFAs can alter the expression of neurotrophic factors, including brain-derived neurotrophic factor (BDNF), which are critical for synaptic plasticity as well as for promoting the growth and survival of neurons (Maqsood and Stone, 2016).

Additionally, SCFAs contribute to the therapy and prevention of neurological diseases. For instance, research has shown that the severity of neurovascular and neurodegenerative illnesses like Alzheimer's and Parkinson's may be correlated with changes in SCFA production brought on by dysbiosis, or an imbalance of gut flora (Goyal et al., 2021; Jia et al., 2024). As a result, dietary changes and probiotics that target the production of SCFAs present a viable therapeutic strategy for promoting neuroplasticity and preventing degeneration.

Beyond SCFAs, gut microbiota-derived metabolites play multifaceted roles in neuroplasticity. Tryptophan metabolism, mediated by gut bacteria, yields key neuroactive compounds such as indole derivatives (e.g., indole-3-propionic acid) and kynurenine pathway intermediates, which modulate serotonin synthesis, glutamatergic signaling, and neuroinflammation which is critical for synaptic plasticity and implicated in depression and neurodegenerative diseases (Gao et al., 2020). The gut microbiota also influences the kynurenine-to-tryptophan ratio, which affects neuroprotective (e.g., kynurenic acid) and neurotoxic (e.g., quinolinic acid) outcomes in the brain (Wang et al., 2019). Lactate, produced by bacterial fermentation (e.g., *Lactobacillus*), enhances neuroplasticity by upregulating brain-derived neurotrophic factor (BDNF) and promoting synaptic strength via N-methyl-D-aspartate receptor activation (a type of ionotropic glutamate receptor that plays a critical role in synaptic plasticity, learning, and memory). Exercise-induced lactate has been linked to increased BDNF levels, supporting neurogenesis and cognitive function (Müller et al., 2020). Polyamines like spermidine, derived from gut microbes, delay brain aging by inducing autophagy, improving mitochondrial function, and upregulating neurotrophic factors (e.g., Nerve growth factor, brain-derived neurotrophic factor) in aging models. Spermidine also reduces oxidative stress and inflammation, preserving synaptic proteins such as postsynaptic density-95 and postsynaptic density-93 (Xu et al., 2020).

Moreover, Microbial phenolic compounds (e.g., 4-ethylphenylsulfate) and aromatic metabolites (e.g., indole-3-aldehyde) exhibit antioxidant and anti-inflammatory properties, influencing mood and cognition. These metabolites interact with aryl hydrocarbon receptors to modulate neuroimmune responses (Ahmed et al., 2022). Secondary bile acids (e.g., deoxycholic acid), produced by bacterial modification of primary bile acids, indirectly affect brain function by regulating blood-brain barrier integrity and systemic inflammation. Dysregulation of bile acid metabolism is linked to neuroinflammation in conditions like Alzheimer's and Parkinson's diseases (Calzadilla et al., 2022).

Collectively, these metabolites highlight the gut microbiome's broad biochemical influence on neuroplasticity, spanning neurotransmission, neuroprotection, and immune modulation.

#### 2.3.2 Immune system modulation

The immune system is strongly influenced by gut bacteria, and this relationship has a major impact on brain function. The maturation and activation of immune cells that affect neuroinflammatory processes are regulated by gut bacteria. Anxiety, sadness, and cognitive impairment are among the neuropsychiatric disorders that have been linked to chronic inflammation (Chesnokova et al., 2016). The production of cytokines, which are immune signaling molecules involved in both systemic and central nervous system (CNS) inflammation, can be influenced by the microbiota.

Research indicates that whereas dysbiosis frequently results in the release of pro-inflammatory cytokines, which can impair neuroplasticity, a balanced gut microbiome encourages anti-inflammatory immune responses (Fung et al., 2017). Notably, research has demonstrated that microbial strains can increase the generation of regulatory T cells (Tregs), which are essential for limiting overreactions to inflammation that may harm brain circuits. Knowing how the microbiota affects immune signaling pathways helps us understand how microbial therapies can lessen the harm that inflammation causes to neuroplasticity.

#### 2.3.3 Neurotransmitter production

Important neurotransmitters such as serotonin, dopamine, and gamma-aminobutyric acid (GABA), which are essential for mood control and cognitive flexibility, are synthesized by gut flora. The gastrointestinal system produces around 90% of the body's serotonin, and gut microbes affect the amount and accessibility of this neurotransmitter to the brain. For example, it has been demonstrated that certain strains of Bifidobacterium and Lactobacillus increase the synthesis of serotonin by altering the metabolism of tryptophan (Strandwitz, 2018).

Furthermore, gut flora also affects the amounts of another neurotransmitter, dopamine. The production of dopamine and norepinephrine precursors by specific bacteria points to a possible mechanism via which the microbiota may influence mood and cognitive processes. Certain gut bacteria also create GABA, the brain's main inhibitory neurotransmitter, which connects gut health to anxiety control and mental wellness in general. It has been demonstrated that dysbiosis results in changed concentrations of these neurotransmitters, suggesting a possible way in which the gut microbiota may affect neuroplastic processes (Verma et al., 2024).

#### 2.3.4 Hormonal regulation

Additionally, microbiota is essential for controlling hormones that have a direct impact on neuroplasticity. For example, neurogenesis and synaptic plasticity are known to be impacted by the hormone cortisol, which is produced in response to stress. Prolonged stress and high cortisol levels can impair cognitive abilities and exacerbate anxiety and depression. According to research, the hypothalamic-pituitary-adrenal (HPA) axis is one of the methods by which the gut microbiota might influence cortisol levels (Vyas et al., 2016).

Dysbiosis can cause dysregulated HPA axis activity, which raises cortisol levels and has a detrimental effect on brain function. On the other hand, by reducing cortisol release and boosting resistance to stress-induced alterations in neuroplasticity, a balanced gut microbiota seems to promote a healthy stress response (Frankiensztajn et al., 2020). Thus, promoting hormonal balance and its positive impacts on neuroplasticity may be possible by addressing gut health through diet, probiotics, and lifestyle modifications.

Within pathways connected by microbial metabolites, immunological modulation, neurotransmitter production, and hormonal regulation, the complex interaction between the gut microbiota and neuroplasticity takes place. Assessing these processes presents a viable path for treatment approaches meant to promote gut health via brain health. Integrative strategies utilizing nutrition and microbial therapy will become essential in treating neurological and psychological diseases as research continues to reveal the intricacies of the microbiota-gut-brain axis (Vodička et al., 2018).

### 2.4 Evidence from animal and human studies

#### 2.4.1 Animal studies

There has been compelling recent evidence from animal studies that the gut microbiome plays a major role in neuroplasticity. For example, the work of Zheng et al. (2016) revealed that germ-free mice, which lack a gut microbiome, exhibited considerable changes in synaptic plasticity; a fundamental aspect of learning and memory. As compared to mice that took a diverse microbiota, the germ-free mice show cognitive deficits. They also correlated these behavioral changes to disruptions in brain-derived neurotrophic factor (BDNF) signaling, an essential protein for neuron survival and growth, as well as the backbone of neuroplasticity (Zheng et al., 2016).

Hsiao et al. (2013) further studied what particular gut microbiota contribute to behavior in another pivotal work. When a variety of beneficial microbiota were introduced to germ-free mice, anxiety-like behavior showed a marked reversal. The cause for this change is thought to be the microbiota's ability to modulate the functioning of the hypothalamic-pituitary-adrenal (HPA) axis, which is an important stress response system (Hsiao et al., 2013).

Compelling evidence from animal models demonstrates that gut microbiota influence neuroplasticity through four interconnected mechanistic pathways. Microbial metabolites like lactate (produced by *Lactobacillus* spp.) enhance hippocampal synaptic plasticity and neurogenesis via NMDA receptor-dependent BDNF upregulation in mice, directly linking bacterial fermentation products to learning and memory consolidation (Müller et al., 2020). Preclinical studies in animal models have demonstrated that gut dysbiosis, which is a disruption in the composition and function of the intestinal microbiota, plays a significant role in modulating key mechanisms associated with Alzheimer's disease (AD), including neuroinflammation, blood–brain barrier integrity, and neurotransmitter homeostasis. These alterations may contribute to both the initiation and progression of AD pathology. Notably, emerging microbiota-targeted interventions, such as probiotics, prebiotics, and fecal microbiota transplantation, have shown potential in ameliorating AD-related neurodegenerative processes (Yang et al., 2024).

Murine models have demonstrated marked alterations in hippocampal neurochemistry and amino acid levels between germ-free (GF) and specific pathogen-free (SPF) mice. GF mice show significantly reduced concentrations of key amino acids, including L-phenylalanine, L-arginine, L-alanine, L-isoleucine, L-leucine, L-glutamine, L-valine, and γ-aminobutyric acid (GABA), compared to SPF controls. Furthermore, elevated expression of reactive microglial markers and increased synaptic density have been observed in the GF hippocampus, suggesting impaired synaptic pruning due to dysregulated microglial activity and overexpression of synaptogenic genes. These changes may contribute to the formation of functionally aberrant synapses. Notably, the same studies underscore the role of *Bifidobacterium* species in supporting the maturation of functional hippocampal circuits (Mitra et al., 2023). Furthermore, hormonal signaling pathways, particularly involving the hypothalamic-pituitary-adrenal (HPA) axis are heavily modulated by gut microbes. It was reported that the gut microbiota affects circulating corticosterone levels in rodents under chronic stress, which in turn affects hippocampal neuroplasticity through glucocorticoid receptor-mediated pathways (Cavaliere and Traina, 2023).

Finally, the gut microbiome plays a vital role in brain development and function through bidirectional gut-brain communication. Alterations in microbial composition are increasingly linked to neuropsychiatric disorders such as autism, depression, and schizophrenia. Evidence from large-scale studies and model animals supports these associations, and microbiome-targeted therapies, including probiotics and fecal transplantation, show promise for future prevention and treatment strategies (Lee et al., 2025). These studies find that the gut microbiome plays an intimate role in shaping brain pathways and cognitive functions (Table 2)

**Table 2:** Evidence from animal and human studies supporting the role of gut microbiota in neuroplasticity.

**Study type**	**Key findings**	**Implicated mechanisms**	**References**
Animal models	Germ-free mice show significant cognitive deficits, impaired memory, and learning, alongside reduced hippocampal BDNF expression, disrupted synaptic plasticity, and abnormal spine morphology.	BDNF signaling impairment Microglial dysfunction HPA axis overactivation Neurotransmitter imbalance	Delgado-Ocaña and Cuesta, 2024
Animal models	*Lactobacillus* and *Bifidobacterium* reduce anxiety/depressive behaviors and improve memory.	Neurotransmitter modulation (GABA, serotonin), HPA axis regulation.	Hsiao et al., 2013; Huang and Wu, 2021
Animal models	SCFAs (butyrate/propionate) enhance synaptic plasticity; lactate upregulates BDNF/NMDA-R signaling.	BDNF signaling, NMDA receptor activation, histone deacetylase inhibition.	Silva et al., 2020; Müller et al., 2020
Animal models	High-fat diets induce neuroinflammation and memory impairment; chronic stress increases vulnerability via microbiota shifts.	Neuroinflammation, oxidative stress, HPA axis hyperactivity.	Shi et al., 2020; Cavaliere and Traina, 2023
Animal models	FMT from aged donors impairs spatial learning; FMT from healthy donors rescues cognitive deficits.	Altered synaptic plasticity proteins, neurotransmission.	D'Amato et al., 2020; Yang et al., 2024
Animal models	*Bifidobacterium* spp. and *Akkermansia muciniphila* promote hippocampal maturation and BDNF production.	BDNF upregulation, immune modulation.	Mitra et al., 2023; Molska et al., 2024
Human studies	Altered microbiota in MDD, ASD, ADHD, Alzheimer's, and Parkinson's vs. healthy controls. Reduced SCFA producers common.	Inflammation, neurotransmitter imbalance, reduced BDNF.	Dash et al., 2022; Kim et al., 2021
Human studies	Probiotics (*Lactobacillus/Bifidobacterium*) reduce depression/anxiety symptoms and improve cognitive flexibility in older adults.	GABA/serotonin modulation, HPA axis regulation.	Kim et al., 2021; Cryan and Dinan, 2012
Human studies	Synbiotics enhance verbal memory in older adults; improve attention and neural processing in children.	Tryptophan metabolism modulation, reduced neurotoxicity (quinolinic acid).	Kim et al., 2021; Mosquera et al., 2024
Human studies	FMT resolved *C. difficile* infection and significantly improved cognition in an Alzheimer's patient.	Microbial restoration, reduced inflammation.	Park et al., 2021
Human studies	Mediterranean/high-fiber diets improve microbiome diversity and cognition; high fat/protein vs. carbs linked to mood.	SCFA production, anti-inflammatory effects.	(Boccardi et al., 2017; Martin et al., 2023)
Human studies	*Ruminococcaceae/Firmicutes* correlate with negative emotions; butyrate producers linked to better emotion regulation in children.	Tryptophan metabolism, B-vitamin synthesis (B2, B3, B6, B9).	Ke et al., 2023; Faulkner et al., 2024

#### 2.4.2 Human studies

Human research adds to the preponderance of evidence suggesting gut microbiome has a vast impact on neuroplasticity. Dysbiosis (defect in the balance and diversity of gut microbiota) is associated with negative consequences of gut level on brain function, and cognitive development. Dysbiosis has been also implicated in neurodevelopmental conditions such as autism spectrum disorder (ASD), attention deficit hyperactivity disorder (ADHD), and even neurodegenerative disorders such as Alzheimer's and Parkinson's (Dash et al., 2022).

The gut-brain axis is the term for a two-way gut-to-brain communication system via the neuroendocrine-immune pathway. Gut Microbiota generates several metabolites including short-chain fatty acids (SCFAs), which are now known to impact neuroinflammation, synaptic plasticity, and effects on BDNF, a key player in learning and memory (Cryan and Dinan, 2012). The gut microbiota also controls levels of neurotransmitters like serotonin, dopamine, and gamma-aminobutyric acid (GABA) all of which are fundamental for controlling mood and cognitive functions (Dash et al., 2022).

In addition, recent studies have shown that dietary interventions, probiotics, or prebiotics can modulate gut microbiome, and then modulate brain plasticity as well as other mental health outcomes. Here's an example: clinical trials have shown that taking a probiotic can relieve some of the symptoms of depression and anxiety, for example, by restoring balance to the gut microbiome, and improving the gut-brain interconnection network (Cryan and Dinan, 2012). These findings highlight the engagement of the gut microbiota therapeutically as a tractable target to increase neuroplasticity and treat neurological and psychiatric disorders (Table 2).

### 2.5 Microbiota and cognitive functions

**Figure 2:**
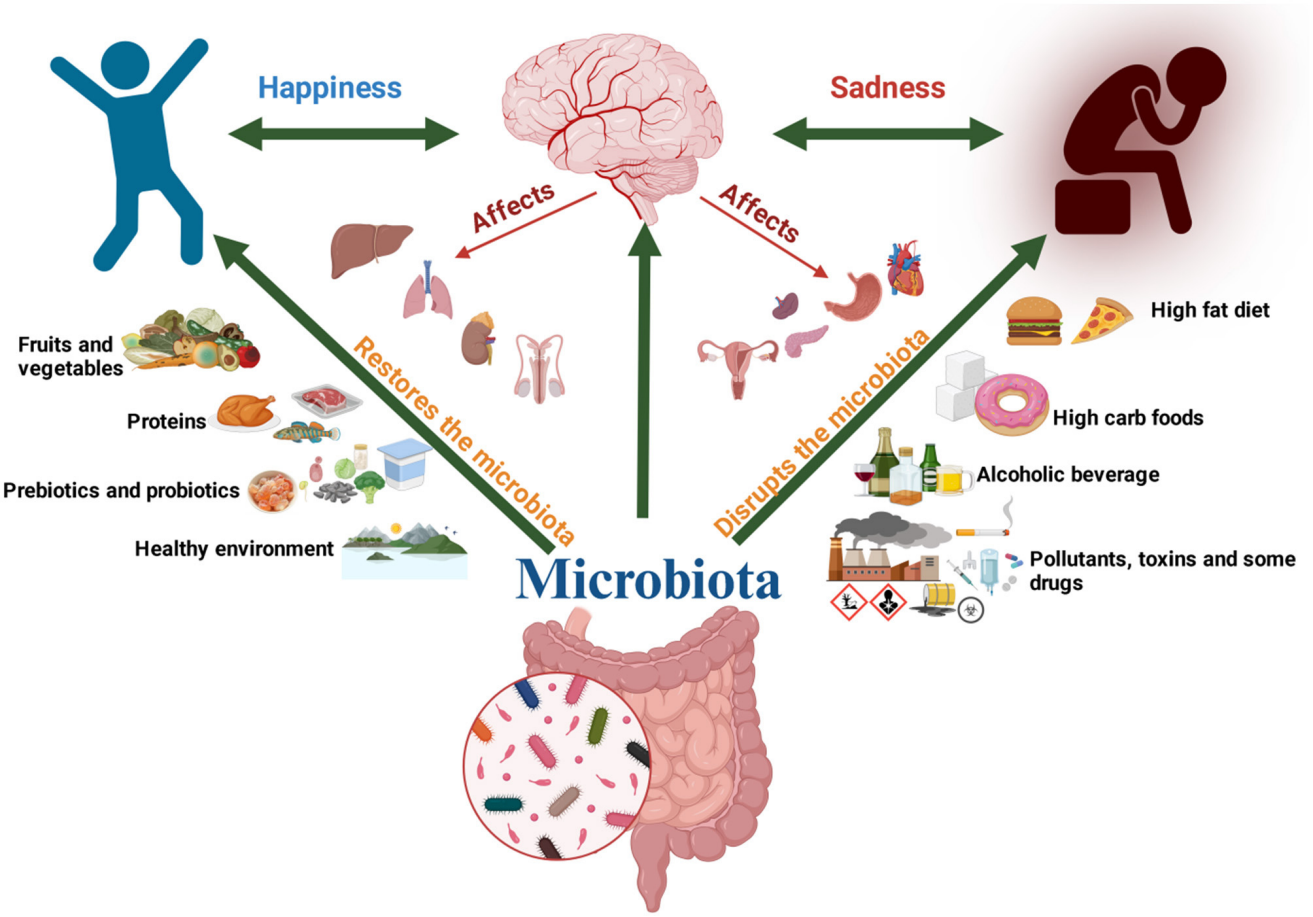
The gut-brain-microbiota axis: impact of diet and conditions on cognitive function.

Recent research has highlighted the critical role of the gut-brain-microbiota axis in regulating and sustaining cognitive functions (Figure 2). In experimental animal models, cognitive abilities, including learning and memory, are typically evaluated through standardized behavioral assays (Gareau, 2014). A study conducted on mice identified a significant association between dietary-induced alterations in bacterial diversity and behavioral changes, suggesting a potential role of gut microbiota diversity in influencing memory and learning (Li et al., 2009). In another study, the microbiota of chicks was investigated, presenting evidence of a feed-forward loop system that connects the microbiota-gut-brain axis to stress and memory function. It was suggested by the findings that maintaining a balanced microbiota may mitigate memory impairments associated with chronic stress. Additionally, the *Alistipes* genus, a gastrointestinal tract bacterium, was identified as a potential biomarker of stress in vertebrates due to its association with the tryptophan metabolism pathway (Kraimi et al., 2022). Fecal microbiota transplantation from aged donor mice has been shown to influence spatial learning and memory in young recipients by modulating hippocampal proteins associated with synaptic plasticity and neurotransmission. Restoring a youthful microbiota could, therefore, enhance cognitive function and address the declining quality of life in older adults (D'Amato et al., 2020). It has been reported that dietary lactate treatment in germ-free mice independently enhanced memory. Additionally, *Lactobacillus* elevated the levels of the neurotransmitter gamma-aminobutyric acid (GABA) in the hippocampus of the mice (Mao et al., 2020).

**Figure 3:**
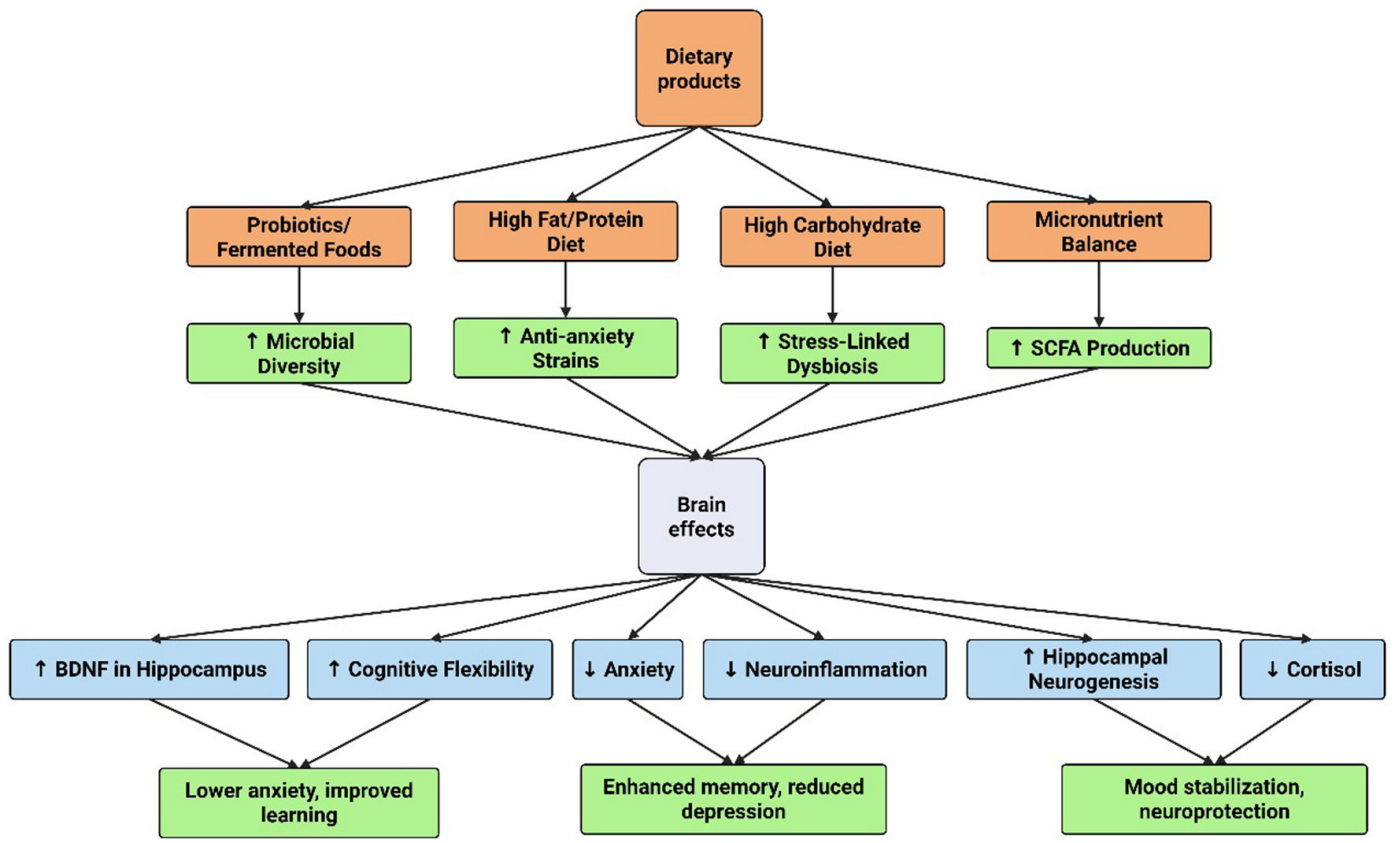
Microbiota and cognitive function.

The majority of studies on the microbiota and cognitive functions, as documented in the literature, have been conducted on experimental animals, with relatively few investigations focusing on humans (Figure 3). However, clinical evidence has supported the hypothesis that intestinal microorganisms undergo changes in individuals with mild cognitive impairment and Alzheimer's disease suggesting a potential role in the pathogenesis of Alzheimer's disease (Zhu et al., 2022). Probiotics have been shown to enhance mental flexibility and reduce stress in healthy older adults, accompanied by alterations in gut microbiota. These findings provide evidence supporting the health-promoting benefits of probiotics as an integral component of a healthy diet for older people (≥65 years) (Kim et al., 2021). A case involving a 90-year-old woman with Alzheimer's dementia and severe *Clostridioides difficile* infection demonstrated notable improvement in cognitive function following fecal microbiota transplantation (FMT). One month after FMT, significant cognitive enhancement was observed compared to her pre-transplantation state, highlighting the potential of microbiota modulation in influencing neurological outcomes (Park et al., 2021).

On the other side, the microbiome influences emotional regulation, mood, and behavior by modulating the gut-brain axis, which impacts neuroplasticity. Microbial metabolites, such as short-chain fatty acids and neurotransmitter precursors, affect synaptic plasticity, neurogenesis, and inflammation in brain regions like the hippocampus and amygdala. These changes alter neural circuits underlying stress responses, mood disorders, and cognitive function (Moloney et al., 2014; Toader et al., 2024). The relationship between gut microbiome composition and these mood disorders is so significant that it is often termed the gut microbiome-brain axis, which is believed to interact with almost every organ in the human body (Cryan et al., 2019). A notable study revealed an inverse association between positive emotions and the relative abundance of *Ruminococcaceae* sp. and *Firmicutes* sp., while negative emotions exhibited a direct correlation with the abundance of these species. At the metabolic pathway level, negative emotions were inversely linked to the biosynthesis of pantothenate, adenosine, and coenzyme A in humans (Ke et al., 2023). In children, a recent study demonstrated that those in the high maladaptive emotion regulation group exhibited significantly lower microbiome, reduced relative abundance of butyrate-producing genera (*Butyricicoccus* and *Odoribacter*), and decreased vitamin synthesis scores for vitamins B2, B3, B6, and B9. These findings suggest that maladaptive emotion regulation skills are associated with alterations in the gut microbiome during early childhood (2–6 years old) (Faulkner et al., 2024). Strong correlations have been reported between dietary patterns and mental health outcomes. Higher consumption of fat and protein has been linked to reduced anxiety and depression, whereas greater carbohydrate intake has been associated with increased levels of stress, anxiety, and depression, as well as changes in gut microbiome diversity (Martin et al., 2023).

### 2.6 Therapeutic implications of microbial influence on neuroplasticity

Recent years have seen a major increase in interest in the connection between gut microbiota and neuroplasticity, which has led to research into treatment approaches that improve cognitive performance via the gut-brain axis (Murciano-brea et al., 2021).

#### 2.6.1 Probiotics and prebiotics

Probiotics are live microorganisms that, when given in sufficient quantities, have been shown to improve neurochemical signaling, reduce inflammation, and affect gut microbiota composition—all of which are critical for neuroplasticity (Gupta and Garg, 2009; Chen et al., 2021). Probiotics are primarily from the *Lactobacillus* and *Bifidobacterium* genera. According to research, probiotics may alter neurotransmitters like GABA and serotonin, encouraging synaptic plasticity and reducing depressive and anxious symptoms (Chen et al., 2021).

Prebiotics, such as fructooligosaccharides (FOS) and galactooligosaccharides (GOS), improve gut-brain communication by specifically promoting the growth and activity of good gut bacteria. Synbiotics, which are a combination of probiotics and prebiotics, improve the gut microbiota's resilience and ability to support cognitive health (Chen et al., 2021). Synbiotics have demonstrated promise in reducing anxiety and depressive symptoms, suggesting that they may be used as therapeutic agents to improve neuroplasticity (Carlessi et al., 2021).

Synbiotic interventions combining Lactobacillus and Bifidobacterium strains with fructooligosaccharides (FOS) have been linked to improvements in memory and cognitive performance in older adults. In a randomized, double-blind, placebo-controlled trial, older participants receiving a synbiotic formulation (including species such as *Lactobacillus paracasei, L. rhamnosus, L. acidophilus*, and *Bifidobacterium lactis* along with FOS) demonstrated enhanced verbal memory and delayed recall compared to placebo (He et al., 2025; Gonçalves et al., 2025).

Experimental studies in animal models support the effects of Lactobacillus paracasei HII01 combined with xylooligosaccharides (XOS). In obese, insulin-resistant rats, supplementation with this synbiotic formulation attenuated gut inflammation, preserved hippocampal synaptic plasticity, reduced oxidative stress, and restored microglial morphology, which are key features of improved neuroplasticity (Thiennimitr et al., 2018).

Additionally, a preliminary clinical investigation involving healthy children supplemented with *L. paracasei* HII01 for 12 weeks demonstrated reduced urinary quinolinic acid (QA) levels, increased 5-HIAA, improved go-accuracy in attention tasks, and EEG changes suggestive of enhanced neural processing and sustained attention (Gonçalves et al., 2025; Mosquera et al., 2024).

#### 2.6.2 Dietary interventions

To improve neuroplasticity and shape gut microbiota, dietary treatments are essential. Numerous dietary patterns, especially those high in fiber, fruits, vegetables, and polyphenols, can help modulate the gut microbiota and support a diverse and balanced microbiome. For example, a diet rich in fiber has been linked to higher levels of the creation of healthy metabolites that promote neurogenesis and brain health, like short-chain fatty acids (SCFAs) (Li et al., 2019).

The Mediterranean diet, which prioritizes whole grains, fruits, vegetables, and healthy fats, has been shown in recent studies to improve microbiome diversity and cognitive performance. By encouraging a healthy gut-brain axis and improving neuroplasticity through many pathways, including lowering oxidative stress and systemic inflammation, this dietary strategy is thought to lower the risk of neurodegenerative illnesses (Boccardi et al., 2017).

Furthermore, certain nutritional ingredients, like omega-3 fatty acids, which are present in fish, have been connected to enhanced neural resilience and cognitive abilities. As part of treatment methods to increase neuroplasticity, it should be a priority to identify and execute dietary regimens that improve gut microbiota diversity and functionality (Zinkow et al., 2024).

### 2.7 Advanced and future therapies

With treatments like fecal microbiota transplantation (FMT), microbiome engineering, and personalized nutrition, the future of therapeutic interventions targeted at enhancing neuroplasticity through the gut microbiome appears bright. Through the transfer of microbiota from a healthy donor to a recipient, FMT has been demonstrated in numerous studies to improve cognitive functioning and restore gut microbiota balance (Table 3). By directly affecting the gut-brain axis, this method has become a viable treatment for several neurological disorders (Tian et al., 2024).

**Table 3:** Therapeutic approaches targeting microbiome for enhancing neuroplasticity.

**Therapeutic approach**	**Mechanism of action**	**Potential benefits**	**Examples**	**Reference**
Probiotics	Introduce beneficial bacteria	Improve cognitive function, reduce neuroinflammation	*Lactobacillus rhamnosus* for anxiety reduction	Xu et al., 2022
Transplantation (FMT)	Transfer of healthy gut microbiota	Potential neuroprotective and mood-regulating effects	Donor fecal transplants for treating depression	Doll et al., 2022
Psychobiotics	Direct impact on mental health through microbiota manipulation	Enhance mood, alleviate anxiety and depression	*Lactobacillus helveticus, Bifidobacterium longum*	De Oliveira et al., 2023

Using a variety of methods, such as genetically modified organisms, microbiome engineering aims to modify the composition of the gut microbiota. Through their metabolites and signaling capacities, this new research seeks to create certain microbial strains that may improve cognitive function and resistance to neurodegenerative illnesses (Missiego-Beltrán and Beltrán-Velasco, 2024).

The link between diet and gut health can be optimized by personalized nutrition approaches that consider individual differences in microbiota composition, dietary practices, and lifestyle factors (Kolodziejczyk et al., 2019). These treatments are designed to optimize advantages unique to a person's microbiome, which may result in better neuroplasticity and cognitive function. These creative approaches demonstrate the versatility of recent neuroscience and nutrition research, highlighting the necessity of investigating the potential role that microbial modulation may play in therapeutic interventions aimed at promoting neuroplasticity (Loh et al., 2024).

### 2.7 Research gaps

#### 2.7.1 Research gaps in mechanistic understanding

The complete comprehension of the underlying mechanisms that explain how gut microbes influence neuroplasticity is still absent. Even, direct interconnections between microbes in the gut and brain circuitry are still primarily unidentified, although some present research trying to evaluate the secondary pathways which involve the chemicals and immune responses. Still, there is a lack of studies that focus on direct interactions between gut microbes and neural circuits. There are some complexity available in the neural circuits. As we all know the central nervous system (CNS) is comprised of complex structures in which different variables influence the mechanism of neuroplasticity, especially synaptic strength, development of neurons, and myelination. Discovering how specific microbial elements interact with multiple procedures, the scientist needs to explore some creative ideas (experimental designs) that are capable of eliciting these complex connections. Individual variability in the composition of the gut microbiota makes it challenging to discover generic mechanisms. Diet, genetics, and impacts from the environment all lead to significant variations in an individual's microbial ecosystems, making it harder to recognize accurate correlations that universally affect the process of neuroplasticity (Murciano-brea et al., 2021).

#### 2.7.2 Methodological challenges

- Study Design Limitation: Animal models, particularly rodents, differ significantly from humans in multiple physiological aspects.
- Gut Topology and Function: The physiology and the method or the operation process of the intestinal tract differ from species to species. As an example, rodents have a simpler gut structure than humans comparably, which can influence the process of the microbes to deal with gut tissues and systemic signaling pathways.
- Immune Systems Variability: The composition and function of rodents' immune systems are technically different in a wide range of aspects from the ones found in humans. These variations can be a reason for the modifications and the actions in how immune responses can be triggered by microbial presence, thus skewing results when applying conclusions from animal studies to situations involving humans.
- Cognitive differences: Rodents do not entirely replicate human brain functions or especially behaviors. As a result, findings evidence of neuroplasticity from rodent models may not precisely represent human experiences or neurological disorders.

Need for Advanced Techniques: Innovative technologies are required mostly for the research purpose of this topic, as this will help to generate immediate detection of microbial consequences for brain activity. In this regard, Optogenetics and advanced imaging tools might be useful or we can say a game changer in the process of discovering direct connections between gut bacteria and brain circuits.

### 2.8 Future research directions for gut-brain axis study

- Holistic Approaches: Multi-integrative methodologies that span distinct biological frameworks (neuroimmune, metabolic) are needed for a greater understanding of the gut-brain axis. Future research or invention needs to concentrate on researching targeted interventions, such as probiotics or modifications to the diet, which can improve neuroplasticity and also help to treat neurological disorders by modifying gut microbiota modulation (Hasan Mohajeri et al., 2018).
- Longitudinal Study: Long-term investigations include studying how changes in gut microbiota all throughout time can link with neuroplasticity modification and altering mental health implications. And, this could provide a beneficial understanding of preventive treatments for neurodevelopmental disorders (Verma et al., 2024).

### 2.9 Limitations

The use of simplistic animal models, especially germ-free (GF) along with particular pathogen-free (SPF) animals, in research on the microbiota of the digestive tract and its effects on neuroplasticity has different drawbacks that make it harder to connect the findings to human systems. While these models have allowed us to enhance our understanding of microbial effects in the brain, they might not accurately represent the intricacies of human microbiota interactions with neuronal networks. Here's an in-depth investigation of these limitations:

- Simplifying Biological Systems for research purpose: Germ-free and gnotobiotic animal models provide a controlled setting for exploring the effects that specific populations of microbes on the physiology of their hosts. However, these models inherently simplify the biological procedures involved in the research and they are not fully representative of the complexity of the host's microbiota and environment.
- Absence of Commensal Microbiota: GF rodents do not have the wide assortment of microbes that are found in a healthy gut. This absence inhibits researchers from witnessing the complex configuration of microbiome interactions with the immune system of the patient, metabolic pathways, and neural networks in the brain, which is important for understanding the different aspects of human health (Moysidou and Owens, 2021).
- Oversimplified interactions: The communications between microbes present in the gastrointestinal tract and host systems are highly dynamic and dependent upon context. GF models cannot reproduce the complex feedback systems that make up a natural microbiome, allowing us with a not sufficient grasp of how microbial diversity operates (Ambrosini et al., 2019).

## 3 Conclusion

The intricate relationship between the gut microbiota and neuroplasticity highlights the profound impact of intestinal microorganisms on brain function, cognition, and mental health. Through mechanisms such as microbial metabolite production, immune system modulation, neurotransmitter synthesis, and hormonal regulation, the gut-brain axis plays a pivotal role in shaping neural plasticity. Dysbiosis has been linked to various neurodevelopmental and psychiatric disorders, underscoring the importance of maintaining a balanced microbiome for optimal brain health. Emerging evidence suggests that microbiota-targeted interventions, including probiotics, prebiotics, dietary modifications, and physical exercise, offer promising therapeutic strategies for enhancing neuroplasticity and mitigating neurological and psychological disorders. Future research should focus on elucidating the direct microbial-neuronal interactions and developing personalized microbiome-based therapies. Understanding and harnessing the microbiome's potential could revolutionize approaches to mental health and cognitive enhancement, paving the way for novel, non-invasive treatment strategies.
